# Intraventricular Injection of Human Dental Pulp Stem Cells Improves Hypoxic-Ischemic Brain Damage in Neonatal Rats

**DOI:** 10.1371/journal.pone.0066748

**Published:** 2013-06-14

**Authors:** Cheng-zhi Fang, Yu-jia Yang, Qin-hong Wang, Yue Yao, Xiao-ying Zhang, Xue-hua He

**Affiliations:** 1 Department of Pediatrics, Xiangya Hospital, Central South University, Changsha, China; 2 Department of Neonatology, Wuhan Children's Hospital, Wuhan, China; University of Queensland, Australia

## Abstract

**Objective:**

To investigate the effect of intraventricular injection of human dental pulp stem cells (DPSCs) on hypoxic-ischemic brain damage (HIBD) in neonatal rats.

**Methods:**

Thirty-six neonatal rats (postnatal day 7) were assigned to control, HIBD, or HIBD+DPSC groups (n = 12 each group). For induction of HIBD, rats underwent left carotid artery ligation and were exposed to 8% to 10% oxygen for 2 h. Hoechst 33324-labeled human DPSCs were injected into the left lateral ventricle 3 days after HIBD. Behavioral assays were performed to assess hypoxic-ischemic encephalopathy (HIE), and on postnatal day 45, DPSC survival was assessed and expression of neural and glial markers was evaluated by immunohistochemistry and Western blot.

**Results:**

The HIBD group showed significant deficiencies compared to control on T-maze, radial water maze, and postural reflex tests, and the HIBD+DPSC group showed significant improvement on all behavioral tests. On postnatal day 45, Hoechst 33324-labeled DPSC nuclei were visible in the injected region and left cortex. Subsets of DPSCs showed immunostaining for neuronal (neuron-specific enolase [NSE], Nestin) and glial markers (glial fibrillary acidic protein [GFAP], O4). Significantly decreased staining/expression for NSE, GFAP, and O4 was found in the HBID group compared to control, and this was significantly increased in the HBID+DPSC group.

**Conclusion:**

Intraventricular injection of human DPSCs improves HIBD in neonatal rats.

## Introduction

Hypoxic-ischemic encephalopathy (HIE) is caused by partial or complete anoxia, a reduction of cerebral blood flow, or a temporary occlusion. HIE is the most common cause of brain injury in fullterm newborns during the perinatal period [1]. The effectiveness of stem cell transplant for HIE treatment has been reported in animal models, and clinical trials are currently being performed (reviewed in [2]–[4]). Cells used in animal models include, but are not limited to, embryonic stem cells, neural stem cells, bone marrow mesenchymal stem cells, and umbilical cord blood stem cells. In terms of use in humans, some of these stem cells are associated with issues in terms of limited donor sources, host-graft rejection, expense, and ethical concerns. Stem cells such as umbilical cord blood stem cells and mesenchymal stem cells in the bone marrow are derived from the mesoderm. The ability of mesodermal stem cells to eventually differentiate to ectodermally derived neurons remains uncertain.

Stem cells derived from dental tissues, such as dental pulp or follicle cells, have properties of neural stem cells as well as mesenchymal stem cells and have been characterized extensively (reviewed in [5]) and recently hypothesized to be potentially useful in cellular therapy for cerebral ischemia [6], [7]. Dental stem cells can be readily obtained (ie, from routine dental procedures such as removal of impacted third molars) and have been shown to possess properties similar to neural stem cells and mesenchymal stem cells [6]. In addition to neuroprotective functions [8] and modulating the activities of host neuronal stem cells [9], dental pulp stem cells (DPSCs) undergo neuronal differentiation *in vitro* and *in vivo*, under appropriate conditions [10]. The reported immunosuppressive properties of DPSCs may also make them attractive for use in allogeneic transplants [11]. The aim of the present study was to determine if intraventricular injection of human DPSCs can reverse functional aspects of HIE in a neonatal rat model.

## Results

### (I) Behavioral assessments

#### (i) T-maze test

**Figure 1:**
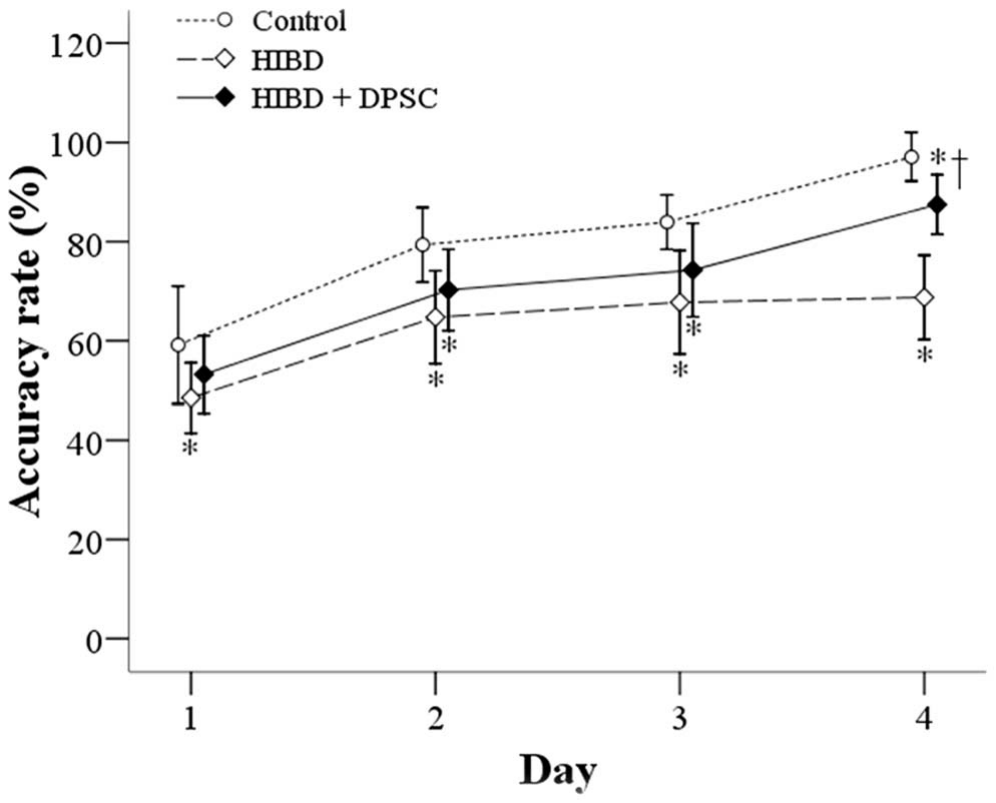
Summary for accuracy (%) in the T-maze test. Data are presented as mean and standard deviation. *Significant difference compared to control group. †Significant difference compared to HIBD group (n = 12 in the control group, n = 10 in the HIBD and HIBD+DPSC groups). DPSC: dental pulp stem cells; HIBD: hypoxic-ischemic brain damage.

Accuracy among the 3 groups increased continuously during the 4 test days. On day 1, the HIBD group showed significantly less accuracy than the control group (48.5% vs. 59.2%, P = 0.0127) (Figure 1). Although the HIBD+DPSC group showed apparent greater accuracy than the HIBD group on day 1, this difference did not reach statistical significance. On days 2 and 3, the HIBD group continued to show significantly less accuracy than the control group (day 2: 64.8% vs. 79.4%, P = 0.0003; day 3: 67.8% vs. 84.0%, P = 0.0001). Again, although the HIBD+DPSC group showed apparent greater accuracy than the HIBD group on days 2 and 3, this difference did not reach statistical significance. The HIBD+DPSC group still showed less accuracy than the control group (day 2: 70.3% vs. 79.4%, P = 0.0163; day 3: 74.3% vs. 84.0%, P = 0.0127). However, on day 4, the HIBD group showed significantly less accuracy than the control group (68.8% vs. 97.1%, P<0.0001), and accuracy was improved in the HIBD+DPSC group compared to the HIBD group (87.5% vs. 68.8%, P<0.0001).

#### (ii) Radial water maze test

Compared to the control group, the HIBD group showed more errors (3.30 vs. 2.08, P = 0.015) and repetitions (2.20 vs. 0.50, P<0.0001), as well as longer water-seeking time (80.60 vs. 53.42 seconds, P<0.0001) (Table 1). Compared to the HIBD group, the HIBD+DPSC group showed significant improvement in errors (2.00 vs. 3.30, P = 0.013), repetitions (1.00 vs. 2.20, P = 0.0007), and water-seeking time (60.10 vs. 80.60 seconds, P<0.0001) (Table 1). No significant differences in errors, repetitions, or water-seeking time were observed between the control and HIBD+DPSC groups.

**Table 1:** Radial Water Maze Test Results for the 3 Groups in This Study.

	Control(n = 12)	HIBD(n = 10)	HIBD+DPSCn = 10)	P value
Water-seeking time (seconds)	53.42 (4.52)	80.60 (12.57)*	60.10 (8.28)†	<0.0001
Errors	2.08 (1.24)	3.30 (1.16)*	2.00 (0.82)†	0.0199
Repetitions	0.50 (0.52)	2.20 (0.92)*	1.00 (0.67)†	<0.0001

Data are presented as mean (standard deviation).

DPSC: dental pulp stem cells; HIBD: hypoxic-ischemic brain damage.

* Significant difference compared to control group.

† Significant difference compared to HIBD group.

#### (iii) Postural reflex

A significant difference in postural reflex score between groups was observed (P = 0.0001) (Table 2). The postural reflex score in HIBD group was significantly higher than in control group (P<0.001), all rats in the control group showed a postural reflex score of 0. Two rats in the HIBD group showed a postural reflex score of 1, and 6 showed a score of 2. The postural reflex score in HIBD+DPSC group was also higher than in control group (P = 0.01); 4 rats in the HIBD+DPSC group showed a postural reflex score of 1, and 1 showed a score of 2. No significant difference in postural reflex score was observed between the HIBD and HIBD+DPSC groups.

**Table 2:** Postural Reflex Scores Among the 3 Groups is This Study.

		Control(n = 12)	HIBD(n = 10)	HIBD+DPSC(n = 10)	P value
Postural reflex score	0	12 (100.0%)	2 (20.0%)	5 (50.0%)	0.0001
	1	0 (0.0%)	2 (20.0%)	4 (40.0%)	
	2	0 (0.0%)	6 (60.0%)	1 (10.0%)	

DPSC: dental pulp stem cells; HIBD: hypoxic-ischemic brain damage.

### (II) Immunofluorescence

**Figure 2:**
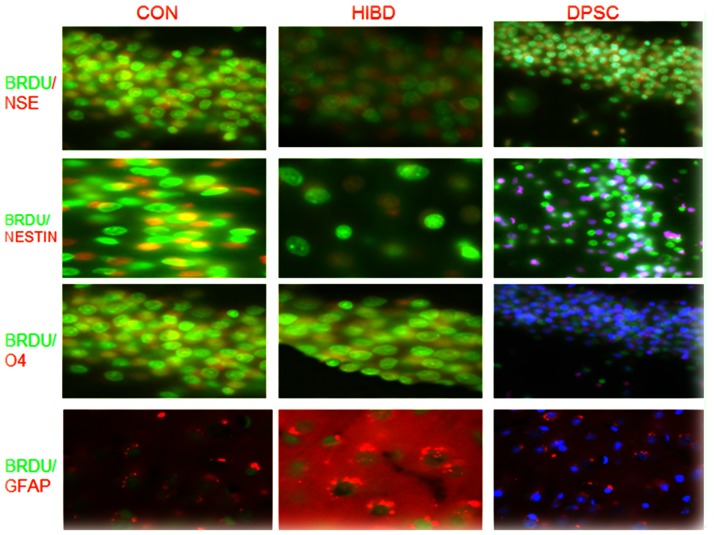
In vivo DPSC differentiation at 37 days after intraventricular injection (300×). The subventricular zone is shown. BrdU: bromodeoxyuridine; CON: control; DPSC: dental pulp stem cells; GFAP: glial fibrillary acidic protein; HIBD: hypoxic-ischemic brain damage; NSE: neuron-specific enolase; O4: oligodendrocyte marker O4.

Hoechst 33324 was used to label DPSC nuclei at the time of injection. At 42 days of age (37 days after injection), frozen sections were prepared, and immunofluorescence staining for NSE, nestin, O4, and GFAP (all red) was performed (Figure 2). BrdU staining is shown as green fluorescence. Percentages of Hoechst 33324-labeled DPSCs showing immunopositivity were 23.70% ± 2.23% for NSE, 17.70%±1.19% for O4, and 13.72%±2.12% for GFAP.

### (III) Immunohistochemistry

Compared to the control group, the HIBD group showed fewer NSE-positive cells (123.60 vs. 278.58, P<0.0001), fewer GFAP-positive cells (37.00 vs. 94.25, P<0.0001), and fewer O4-positive cells (57.80 vs. 124.00, P<0.0001) (Table 3). After DPSC injection, the number of NSE-, GFAP-, and O4-positive cells increased compared to the HIBD group (NSE: 211.60 vs. 123.60, P<0.0001; GFAP: 71.50 vs. 37.00, P<0.0001; O4: 90.40 vs. 57.80, P<0.0001), but these values were still less than those in the control group (all P<0.0001).

**Table 3:** Immunohistochemical Results for NSE, GFAP, and O4 in Rat Cerebral Tissues.

	Control(n = 12)	HIBD(n = 10)	HIBD+DPSC(n = 10)	P value
NSE	278.58 (25.40)	123.60 (21.09)*	211.60 (24.95)* †	<0.0001
GFAP	94.25 (6.66)	37.00 (6.36)*	71.50 (9.19)* †	<0.0001
O4	124.00 (17.06)	57.80 (6.96)*	90.40 (10.39)* †	<0.0001

Data are presented as mean (standard deviation) of positive cell numbers.

DPSC: dental pulp stem cells; GFAP: glial fibrillary acidic protein; HIBD: hypoxic-ischemic brain damage; NSE: neuron-specific enolase; O4: oligodendrocyte marker O4.

* Significant difference compared to control group.

† Significant difference compared to HIBD group.

We also quantified BrdU-labeled (proliferating) cells co-labeled for neuronal (NSE, nestin), or glial (GFAP, O4) markers in each of the groups. The number of BrdU-positive cells was 224.29±34.67 in the control group, decreased to 118.23±19.38 in the HIBD group, and increased back to 185.47±25.72 in the DPSC group. Similar changes were observed in BrdU-positive neuronal cells (control: 156.62±28.67; HIBD: 72.61±15.69; DPSC: 112.58±26.64) and BrdU-positive glial cells (control: 74.34±17.25; HIBD: 45.29±11.25; DPSC: 74.18±16.42). These results indicated that HIBD decreased proliferation and that DPSC administration was able to ameliorate this to a large extent.

### (IV) Western blot

**Figure 3:**
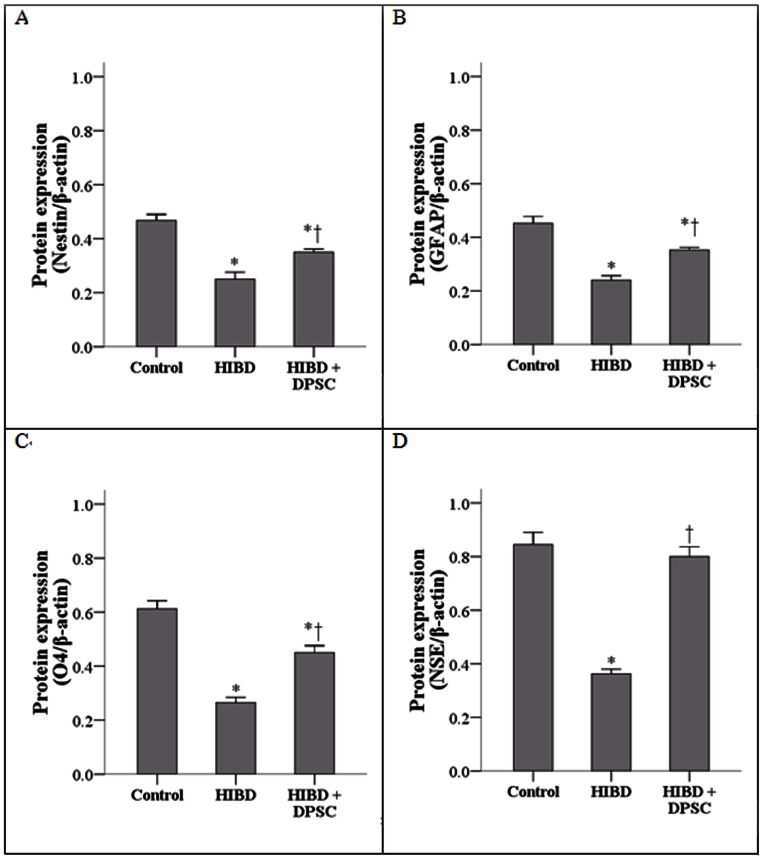
Protein expression levels were performed by Western blot. Four groups are nestin (A), GFAP (B), O4 (C), and NSE (D). Data are presented as mean and standard deviation (n = 4 for each group). for nestin (A), GFAP (B), O4 (C), and NSE (D)*Significant difference compared to control group (P<0.0001). †Significant difference compared to HIBD group (P<0.0001). DPSC: dental pulp stem cells; GFAP: glial fibrillary acidic protein; HIBD: hypoxic-ischemic brain damage; NSE: neuron-specific enolase; O4: oligodendrocyte marker O4.

Protein expression levels for nestin, GFAP, O4, and NSE were significantly decreased in the HIBD group compared to the control group (all P<0.0001) (Figure 3). Injection of DPSCs inhibited these HIBD-induced decreases (all P<0.0001). However, expression levels of nestin, GFAP, and O4 were still less than those in the control group.

## Discussion

Results of the present study showed that intraventricular DPSC injection exerted significant ameliorative effects with respect to HIBD-induced behavioral dysfunction and expression of neural and glial markers in neonatal rats. To our knowledge, this is the first use of DPSCs in this animal model. Establishment of HIBD on postnatal day 7 resulted in significantly decreased ability to perform on the T-maze and the radial arm water maze and showed significant detrimental effects with respect to postural reflex several weeks later (postnatal days 32–42), consistent with previously reported data [14]. Intraventricular injection of DPSCs 3 days after the induction of HIBD showed significant improvements in all 3 behavioral assays, although performance was not back to control levels. These results showed a clear effect of DPSCs on HIBD.

Hoechst 33324-labeled DPSCs were identified in the injected region and left cortex at postnatal day 45, and subsets of DPSCs showed immunostaining for neuronal or glial markers. These results indicated that at least some of the injected cells survived several weeks *in vivo* in rats with neonatal HIBD and that subsets appeared to differentiate along the neuronal and/or glial lineages. Early postnatal induction of HBID resulted in significantly decreased staining/expression for nestin, NSE, GFAP, and O4 several weeks later (postnatal day 45) compared to the control group, and this was significantly ameliorated by DPSC injection. These results showed a clear effect of DPSCs on HIBD-induced expression of neuronal and glial markers. The increases in neural and glial markers in response to DPSC injection could reflect any or all of the following: direct differentiation of injected cells into neurons or glia (as suggested by the subsets of Hoechst 33324-labeled DPSCs showing staining for neuronal or glial markers), promotion of host stem cell proliferation and differentiation into neurons or glia, and/or secretion by DPSCs of factors exerting protective effects on existing neuronal or glial populations. Additional studies are necessary to determine which of these processes occur.

## Materials and Methods

### (I) Reagents

Alpha-minimal essential medium (*α*-MEM), collagenase type I, fetal bovine serum (FBS), dispase, and basic fibroblastic growth factor (bFGF), were purchased from Life Technologies (Grand Island, NY, USA). Glial fibrillary acid protein (GFAP) monoclonal antibody, neuron specific enolase (NSE) monoclonal antibody, nestin monoclonal antibody, rabbit NSE polyclonal antibody, rabbit GFAP polyclonal antibody, Cy3 goat anti-rabbit IgG, Cy3 goat anti-mouse IgG, and fluorescein isothiocyanate (FITC)-labeled goat anti-rabbit IgG were purchased from Santa Cruz Biotechnology (Santa Cruz, CA, USA). O4 monoclonal antibody was purchased from Chemicon (Temecula, CA, USA). Bromodeoxyuridine (BrdU) was purchased from Sigma-Aldrich (St. Louis, MO, USA). Dimethyl sulfoxide (DMSO) was from Xi′an Chemical Reagent Factory (Shaanxi, China). In addition, an immunofluorescence kit (Wuhan Boster Biological Technology, Wuhan, China) and an inverted phase-contrast microscope and camera system (Olympus, Tokyo, Japan) were used.

### (II) Animals and induction of HIBD

This study received institutional review board and institutional animal care and use committee approval, and all experiments were carried out in accordance with *The Guide for the Care and Use of Laboratory Animals* of Institutional Review Board, Xiangya School of Medicine, Central South University, Changsha, China. Thirty-six 7-day-old Sprague-Dawley rats (12–18 g) were provided by the Laboratory Animal Center of Xiangya School of Medicine, Central South University, Changsha, China. Seven-day-old rats are in the peak period of brain development, equivalent to the human neonatal period. Brain damage caused by hypoxia-ischemia at this time is similar to perinatal asphyxia in fullterm children [11]. The rats were randomly assigned to 1 of 3 groups: control (n = 12); hypoxic-ischemic brain damage (HIBD) (n = 12); and HIBD+DPSC (n = 12). The rats were weaned at 21 days of age and fed in separate cages, according to sex.

HIBD was induced on postnatal day 7 according to the method of Rice et al. [12]. The rats were anesthetized with ether, and the left common carotid artery was isolated for double ligation. The middle artery was cut, and the rats were placed in a hypoxic chamber (8% to 10% oxygen) at 37°C for 2 h. To detect new cell proliferation, 2 days before the rats were killed, BrdU (50 mg/kg) was administered intraperitoneally once every 12 h for a total of 4 injections. The rats were killed 4 h after the last injection. Rats in the control group underwent sham surgery; the carotid artery was isolated but was not ligated, and there was no hypoxia exposure.

### (III) Human DPSC culture and preparation for injection

Written consent was obtained in advance from adult patients or from guardians on the behalf of minors/children participants involved in this study. The consent procedure was approved by the Ethics Committees of the Institutional Review Board, Xiangya School of Medicine, Central South University, Changsha, China.

The human DPSC culture method was performed according to Gronthos et al. [13]. Third molars were removed after disinfection from healthy young adults (16–30 years of age) and immediately placed in precooled, incomplete medium containing penicillin (100 U/ml) and streptomycin (100 µg/ml). The teeth were then disinfected with povidone-iodine for approximately 5 min. Phosphate-buffered saline (PBS) containing penicillin (100 U/ml) and streptomycin (100 µg/ml) was used to wash the teeth repeatedly. The dental crown was cut, and the pulp was removed. The dental pulp tissue was cut into pieces with curved microscissors and transferred to centrifuge tubes. A 10× trypsin solution containing 3 mg/ml type I collagenase and 4 mg/ml dispase was added and mixed gently. After digestion at 37°C for 1 h, the solution was mixed to form a single-cell suspension. The cell suspension was passed through a 200 mesh/70 micron cell strainer, centrifuged at 800 rpm at 4°C for 5 min, and the supernatant was removed. The precipitate was mixed with *α*-MEM containing 15% FBS and 2 mM glutamine at 4°C. Cells were placed in a 5% CO_2_/O_2_ incubator, and the medium was replaced every 3 days. Colony formation was observed under a microscope at approximately day 14.

At 80% to 90% confluence, cells were dissociated with trypsin, and precipitates were centrifuged. A volume of 10^6^ cells was resuspended in cryopreservation medium (*α*-MEM containing 15% FBS containing 10% DMSO), placed in cryopreservation tubes, and immediately placed in liquid nitrogen, followed by storage at −80°C until use. Cells were thawed at 37°C, centrifuged to remove the cryopreservation medium, resuspended in fresh medium, and plated in a 10-cm cell culture dish. Cells were passaged at full confluence and used for injection at passage 3 to 5. Before injection, the cells were labeled with 1 *μ*g/ml Hoechst 33324 for 24 h. Labeled cells were collected and stained with 4% trypan blue dye. The viable cell density was adjusted to 5×10^7^/ml, and the cells were placed on ice until injection. We use dental cell-specific markers, such as Stor-1, Col-1, and dentin sialoprotein (DSP) to characterize DPSCs (data not shown).

### (IV) DPSC injection

Rats in the HIBD+DPSC group underwent DPSC injection 3 days after HIBD. Rats were placed in a stereotaxic apparatus under low-temperature anesthesia. The left lateral ventricle (0.5 mm caudal to and 2 mm left of bregma, 2 mm in depth) was used as the injection target. The rats received injection with 2 µl DPSC suspension (10^5^ cells) for more than 5 min. The syringe was left in place for an additional 5 min and was gradually removed. The scalp was sutured and disinfected with 75% ethanol, and the rats were warmed and returned to their cages after complete reawakening.

At 45 days of age (37 days after HIBD), rats (n = 8) were perfused through the heart with 4% paraformaldehyde in 0.1 M PBS, and the brains were collected. Coronal serial sections (16 µm) were cut from 2 mm before to 2 mm after bregma. The sections were frozen at −20°C until use for immunohistochemistry. Hoechst 33324-positive cells were observed under a fluorescence microscope.

### (V) Behavioral assessments

Four rats died during the study (2 in the HIBD group and 2 in the HIBD+DPSC group). Rats in each group (control, n = 12; HIBD, n = 10; HIBD+DPCS, n = 10) were subjected to double-blind behavioral assessments as described below.

#### (i) T-maze test

The T-maze test was performed with rats at 32 days of age. The T-maze consisted of a trunk (60-cm length, 10-cm width, 20-cm height) and the 2 choice arms and (30-cm length, 10-cm width, 20-cm high). The reward (sunflower seeds) was placed in a cup. Food intake was restricted (fasting for 6 h) for 3 days before testing, so that body weight dropped to 80% to 90% of normal.

Two days before testing, the rats were allowed to explore the maze for a few minutes and to find the food. Testing was performed in a quiet room with stable environmental conditions. Before testing, each rat was handled and allowed to adapt to the maze and to find the food bowl for 10 min. Each trial consisted of 2 parts, a forced run and a test run. For the forced run, 1 of the maze arms was arbitrarily closed with a wooden gate, so that the rat could only enter 1 arm to obtain food. After feeding, the rat was removed, and the bowl was immediately removed and cleaned with alcohol. After 15 min, the gate was removed. For the test run, all arms were open, and the rat was placed in the starting arm. If the rat chose not to enter the previously closed arm, food was rewarded (accurate response). If the rat entered the previously closed arm, food was withheld. Each rat was subjected to 4 trials per day, at 15-min intervals, for 4 days to determine the accuracy rate per day for each group of rats. Training was complete when an average accuracy rate of 85% or greater was achieved. For testing, the time interval between the forced run and test run was extended by 1 min and 3 min to calculate the accuracy rate for each group.

#### (ii) Radial water maze test

The radial maze test was performed with rats at 40 days of age. The maze was made of wood, with a central portion consisting of a platform (30-cm diameter) with 8 distribution arms (50-cm length, 12-cm width) and a hole for water at the end of each arm. Water was restricted for 48 h before the test; drinking water was placed in the cage every evening for 30 min and then removed. Two days before testing, 50 µl of water was placed in each arm hole, and the rat was allowed to explore the maze freely. During the test, only 3 arms contained water. The following parameters were recorded: 1) the time to find the 3 arms containing water (water-seeking time); 2) the number of times each arm with water was entered (repetitions); and 3) the number of times each arm without water was entered (errors). Testing was performed at 1-min intervals 5 times a day for 3 consecutive days to calculate the average water-seeking time, the number of errors, and the number of repetitions.

#### (iii) Postural reflex

For assessment of postural reflex, each rat was seized and held by the tail 50 cm from the desktop. Healthy rats place both forelimbs toward the desktop (score of 0), whereas rats with damage to left of the cerebral hemispheres place the contralateral limb (right side) in a flexion-like position (score of 1). Then placed the rats on the table and set pressure on the side of back shoulder until the forelimbs straightened. Repeated several times and recorded as abnormal if toward the contralateral limb (right side) of resistance became weakened (score of 2).

### (VI) Immunofluorescence staining for NSE, Nestin, O4, and GFAP

For each group, fresh-frozen sections (as described above) were dried at room temperature. Immunohistochemistry was performed with primary antibody (anti-NSE, anti-nestin, anti-O4, or anti-GFAP monoclonal antibody; all 1∶100 dilution) and Cy3 goat anti-mouse IgG secondary antibody (1∶500 dilution). PBS was substituted for primary antibody as a negative control (data not shown). The numbers of Cy3-positive cells among Hoechst 33324-positive cells in the left hemisphere were counted under a fluorescence microscope (5 nonoverlapping fields for each slice were selected, and the percentage of positive cells was calculated). All the above steps were performed in the dark.

### (VII) Immunohistochemistry

Frozen slices from the caudal-to-bregma sections were incubated at room temperature with 3% H_2_O_2_ for 30 min. After washing 3 times for 5 min each with distilled water, the sections were incubated with normal goat serum (10%) for 1 h at room temperature. The sections were incubated with anti-O4 monoclonal antibody (Santa Cruz, CA, USA) (1∶100 dilution) for 1 h at room temperature, followed by overnight at 4°C. The sections were then incubated with Cy3 goat anti-mouse antibody (1∶500 dilution). A CMIASWIN medical image analysis system (Bio-Rad, Hercules, CA) was used to count O4-positive cells under light microscopy. A total of 500 cells were assessed per field from 10 nonoverlapping fields in the left (lesioned) periventricular and subcortical white matter regions.

### (VIII) Western blot

Sodium dodecyl sulfate-polyacrylamide gel electrophoresis (SDS-PAGE) was performed according to standard procedures. Forty-microliter samples were mixed with 2× SDS loading buffer (100 mM Tris-HCL, pH 6.8, 4% SDS, 20% glycerol, 200 mM dithiothreitol, 2% bromophenol blue) and boiled at 100°C for 5 min. Protein samples (30 µg per well) were run on 10% SDS gels, and proteins were transferred to nitrocellulose membranes at 60 V at 4°C overnight.

For Western blot, each nitrocellulose membrane was placed in blocking solution (2% bovine serum albumin) for 4 h at room temperature on a vortex shaker. After appropriate washes, rabbit nestin, GFAP, O4, and NSE polyclonal antibodies (all 1∶100 dilution) were added and incubated at 4°C overnight. After three 10-min washes with 0.1% Tris-buffered saline (TBS) containing 0.1% Tween 20, horseradish peroxidase-conjugated goat anti-rabbit IgG (1∶500 dilution) was added and incubation for 1 h on a vortex shaker. The membrane was washed 3 times (10-min each) with 0.1% TBS containing 0.1% Tween 20. Protein bands were visualized with 3,3′-diaminobenzidine (DAB). Bands were scanned and digitized with a computer. Western blot for *β*-actin was used as a sample loading control. Imaging software (Quantity one v4.62; Bio-Rad, Life Science, Hercules, CA, USA) was used to analyze band intensity. Relative protein expression was calculated as band intensity/*β*-actin band intensity. Each experiment was run in triplicate.

### Statistical analysis

Data are presented as mean and standard deviation, with the exception of postural reflex score, which is presented as count and percentage. For continuous data, one-way analysis of variance (ANOVA) with Bonferroni posthoc tests for pair-wise groups was performed to test the difference between control, HIBD, and HIBD+DPSC groups. For postural reflex score, Fisher exact test was performed to evaluate postural reflex scores among the groups. A P value less than 0.05 was considered statistically significant. Statistical analyses were performed with SPSS 15.0 (SPSS Inc., Chicago, IL, USA).

## Conclusion

In conclusion, intraventricular injection of human DPSCs improves HIBD in neonatal rats. The effects of DPSC administration on behavioral assays were limited; it will be important to determine if the effects can be increased or augmented by injecting additional DPSCs or extending the time periods of the assays. Potential limitations of the present study include relatively small sample size; a total of 4 rats died during the study period. In addition, we did not include a Control+DPSC group, which would have allowed us to determine whether the numbers of injected cells expressing neuronal/glial markers differed from those in normal brain (Control+DPSC) vs. injured brain (HIBD+DPSC). We also did not quantify apoptosis by terminal deoxynucleotidyl transferase dUTP nick end labeling (TUNEL assay). Even we use dental cell-specific markers, such as Stor-1, Col-1, and dentin sialoprotein (DSP) to characterize DPSCs, DPSCs may still inadequately characterize. Future studies will include more rats per group, to compensate for any loss of animals during the experiments. In addition, statistical significance in the HIBD+DPSC group was seen at day 4 for the T-maze. Future studies will extend this period out to determine if additional significance can be obtained.
